# Improved redox anti-cancer treatment efficacy through reactive species rhythm manipulation

**DOI:** 10.1038/s41598-020-58579-2

**Published:** 2020-01-31

**Authors:** Uma Kizhuveetil, Sonal Omer, D. Karunagaran, G. K. Suraishkumar

**Affiliations:** Department of Biotechnology, Bhupat and Jyoti Mehta School of Biosciences building, Indian Institute of Technology Madras, Chennai, 600036 India

**Keywords:** Chemical engineering, Chemotherapy

## Abstract

Rhythms in the pseudo-steady state (PSS) levels of reactive species (RS), particularly superoxide and hydroxyl radicals, exist in cancer cells. The RS rhythm characteristics, particularly frequency and amplitude, are entrained (reset) by the anticancer compounds/drugs. In this work, we show for the first time that the phase of the RS rhythm at which the drug is added is significantly important in determining the cytotoxicity of anticancer compounds/drugs such as menadione and curcumin, in two different cancer cell lines. Curcumin, the more effective of the two drugs (IC_50_ = 15 µM, SiHa; 6 µM, HCT116) induced reset of superoxide and hydroxyl rhythms from 15.4 h to 9 h, and 25 h to 11 h respectively, as well as caused increases in these radical levels. However, menadione (IC_50_ = 20 µM, SiHa; 17 µM, HCT116) affected only the superoxide levels. Drug treatment at different time points/phase of the RS rhythm resulted in a maximum of 27% increase in cytotoxicity, which is significant. Further, we report for the first time, an unexpected absence of a correlation between the intracellular PSS RS and antioxidant levels; thus, the practice of using antioxidant enzyme levels as surrogate markers of intracellular oxidative stress levels may need a re-consideration. Therefore, the RS rhythm could be a fundamental/generic target to manipulate for improved cancer therapy.

## Introduction

Reactive oxygen species (RS) such as superoxide and hydroxyl radicals seem to be an important set of molecular mediators of the effectiveness of many anticancer therapies; they are also important determinants of the cellular redox status and conditions such as hypoxia^1–7^. RS are also known to regulate cellular rhythms, and the components of the cellular redox system such as glutathione, glutathione reductase and NAD^+^ have been shown to be rhythmic^8–11^. Such rhythms along with their crosstalk with the timekeeping mechanisms control the metabolic, transcriptional and translational machinery in the cells^12,13^. Further, the rhythms and their alterations have been linked to immune gating responses, lipid peroxidation levels as well as to drug resistance observed in some treatments^4,14–19^. However, the rhythms in the pseudo-steady state (PSS) levels of the more fundamental molecules, the RS themselves, have not been reported in the above context, probably due to an incorrect perception of their utility, given their high reaction rates.

The cellular antioxidant levels have been used as an indirect measure of the oxidative stress in the cells^20^, and oxidative stress is caused by an imbalance in the rates of production and consumption of reactive species^21^. The indirect measure seems to arise from an expectation based on the molecular interactions between enzymatic antioxidants and the relevant RS – e.g. superoxide dismutase (SOD) and superoxide. However, the dynamic aspects of the cell system do not seem to be considered in that expectation. For example, the rate constants of the RS reactions are many orders of magnitude higher than the synthesis rates of enzymatic antioxidants through transcription/translation. Also, the presence of a certain basal level of antioxidants does not explain the dynamic relationship between the RS and antioxidants. Here we show that no correlation exists between the temporal intracellular PSS specific levels of SOD and superoxide in untreated or drug treated SiHa or HCT116 cells. It is a reasonably common practice to use the easily measurable antioxidant enzyme levels as a surrogate measure of oxidative stress caused by increased RS levels in cells. The lack of a relationship between RS and the relevant antioxidant in a cancer cell line (mammalian cell), a bacterium and a microalga suggest that the above common practice needs re-consideration.

The pseudo-steady-state levels of RS can be measured using cell-permeable fluorescent dyes. The common approach, however, is to measure the total oxidative capacity of the cells in terms of H_2_DCFDA fluorescence. This approach is inaccurate due to contributions to measured fluorescence by many molecules^21^. However, the use of other dyes to obtain the PSS levels of RS has been shown to be valuable^22^. They also provide the variations in individual PSS RS levels, which we show to be important in terms of drug activity.

In this work, we report the rhythmic nature of temporal variation of PSS RS levels in two cancer cell lines, the cervical cancer cell line SiHa and the colon cancer cell line HCT116. We also report the entrainment (reset) of these rhythms upon treatment with two well-known anticancer agents, menadione and curcumin. The cytotoxicity induced by the drugs was dependent on the time of drug addition. A larger reset of rhythm was associated with higher therapeutic efficacy of the drug, which implies a relationship between cell death and RS rhythm reset. Analyses of the basal and altered rhythms of RS in cancer cells are expected to improve our understanding of stress response and RS homeostasis in cancer treatment.

## Results and Discussion

### Menadione and curcumin differently altered superoxide and hydroxyl radical levels

**Figure 1:**
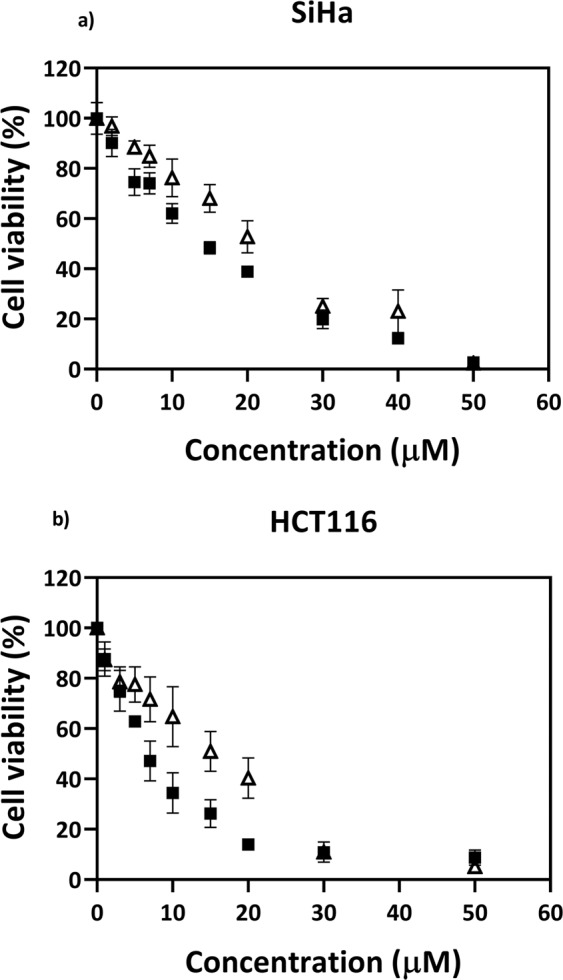
Variation of cytotoxicity of menadione (Δ) and curcumin (■) with concentration on (**a**) SiHa and (**b**) HCT116. The cells were seeded at 1 × 10^4^ cells per well. The cell viability was measured using MTT, 48 h post drug treatment. Values are expressed as mean ± SD, n = 3.

Menadione (vitamin K3) is a synthetic vitamin, which induces superoxide production through a redox cycling mechanism^23^. Curcumin, obtained from *Curcuma longa*, is a polyphenolic compound reported to have pro-oxidant effects^24^. Even though the exact mode of action of curcumin is not well known, both the compounds are known to induce cytotoxicity in cancer cells through induction of RS^25,26^. We determined the cytotoxicity induced by menadione and curcumin in SiHa, a cervical cancer cell line, and HCT 116, a colon cancer cell line, using the MTT assay. It was observed that both menadione and curcumin showed a concentration-dependent cytotoxic effect on both cell lines. However, curcumin was the more effective of the two drugs with an IC_50_ value of 15 µM and 6 µM respectively, in SiHa and HCT116, compared to menadione, which had an IC_50_ of 20 µM and 17 µM, respectively, as shown in Fig. 1a,b.

**Figure 2:**
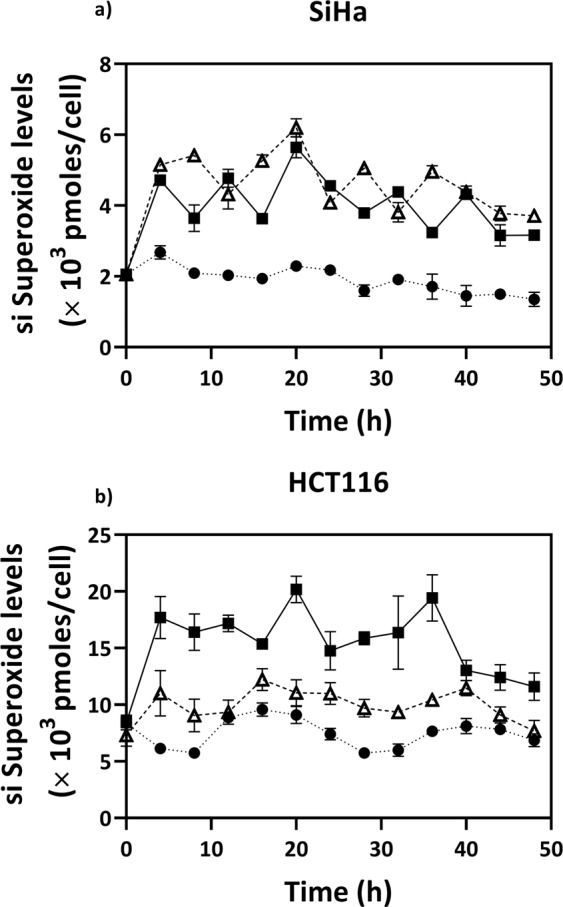
Temporal variations of specific intracellular concentration of superoxide anion radicals (si Superoxide) in untreated (●), menadione treated (Δ) and curcumin treated (■) cells (**a**) SiHa and (**b**) HCT116. Cells were seeded in 6 well plates and synchronized for 24 h in serum free medium. 0 h corresponds to the time of medium change to DMEM with 10% FBS with treated or untreated. Values are expressed as mean ± SD, n = 3.

**Figure 3:**
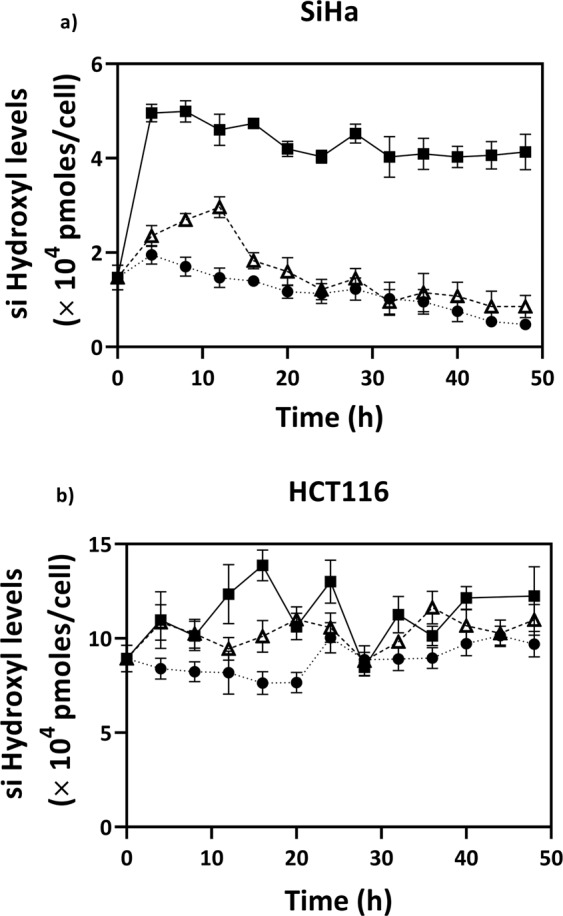
Temporal variations of specific intracellular levels of hydroxyl anion radicals (si Hydroxyl) in untreated (●), menadione treated (Δ) and curcumin treated (■) cells **(a)** SiHa and (**b)** HCT116.  Cells were seeded in 6 well plates and synchronized for 24 h in serum free medium. 0 h corresponds to the time of medium change to DMEM with 10% FBS with treated or untreated. Values are expressed as mean ± SD, n = 3.

Menadione treatment resulted in increased superoxide levels as shown in Fig. 2a,b. The superoxide levels induced in menadione treated SiHa showed a maximum of 3-fold increase compared to the untreated control for the same time point. Menadione induced a 2.2-fold increase in superoxide levels in HCT116 as well. However, menadione did not induce a comparable change in the hydroxyl radical levels in either of the cell lines as can be seen in Fig. 3a,b.

On the other hand, curcumin effected changes in both superoxide (Fig. 2) and hydroxyl (Fig. 3) radical levels. The effect of curcumin on hydroxyl radical levels was more pronounced in SiHa with a maximum 3.4-fold increase as compared to a 1.9-fold increase in HCT116, whereas superoxide levels showed a maximum of about 2.8-fold increase in both the cell lines. These results imply that the mode of action of the two drugs involves mechanisms that alter different types of RS in the cell. The various RS such as hydrogen peroxide, superoxide, hydroxyl, peroxyl, etc., have widely different damage potentials^27,28^. The levels of ‘total ROS’ (ROS- reactive oxygen species) measured through say, H_2_DCFDA, a popular fluorescent probe, may not be an appropriate measure of oxidative stress in such cases^21^.

### The temporal levels of antioxidants and reactive species are not correlated

**Figure 4:**
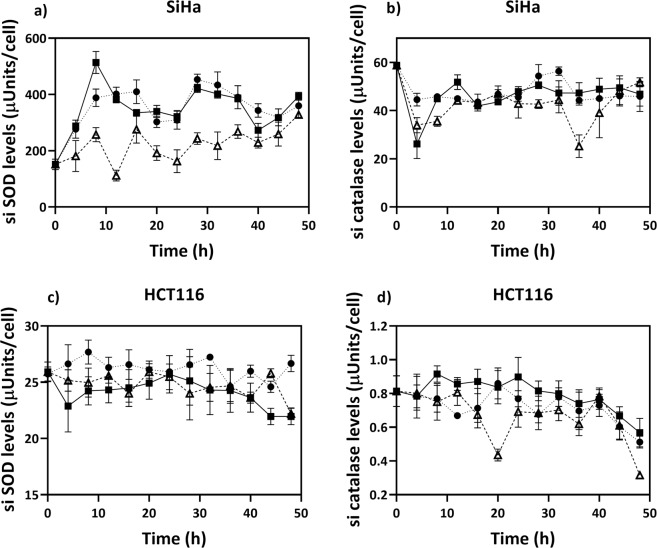
Temporal variations of (**a**) SOD enzymes, (**b**) catalase enzyme in untreated (●), menadione treated (Δ) and curcumin treated (■) SiHa, (**c)** SOD enzymes, (**d**) catalase enzyme in untreated (●), menadione treated (Δ) and curcumin treated (■) HCT116. Cells were seeded in 6 well plates and synchronized for 24 h in serum free medium. 0 h corresponds to the time of medium change to DMEM with 10% FBS with treated or untreated. Values are expressed as mean ± SD, n = 3.

The expression profiles of the antioxidant enzymes are more commonly used to represent the variations of the redox status of the drug-treated cells^20^. Therefore, we decided to check the SOD and catalase temporal profiles in menadione and curcumin-treated SiHa and HCT116. Menadione treated SiHa cells showed lesser levels of SOD as compared to the untreated SiHa cells. Although menadione did not induce any considerable changes in SOD levels in HCT116 (Fig. 4c), it reduced the levels of SOD in the 20 µM menadione treated SiHa by about 75% as compared to the control at 12 h post drug treatment (Fig. 4a). Catalase levels were not significantly affected in either of the menadione treated cells (Fig. 4b,d). The effect of curcumin on SOD or catalase levels were not significant in both the cell lines (Fig. 4).

**Figure 5:**
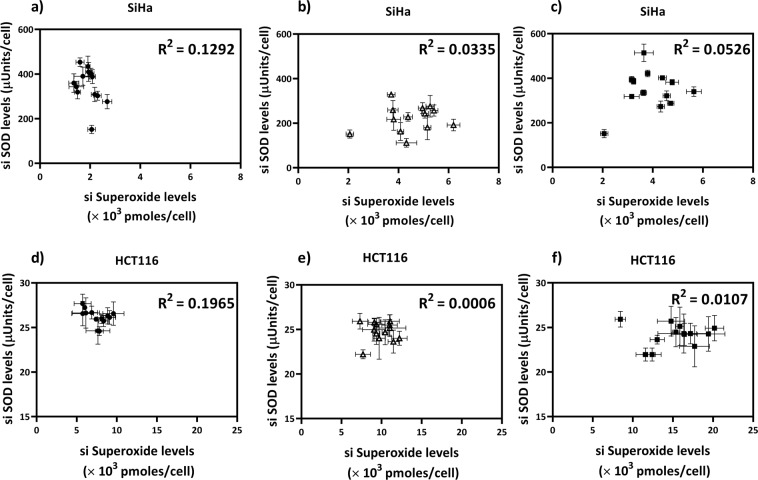
There is no evident correlation between SOD and si superoxide levels in (**a**) untreated (●), (**b**) menadione treated (Δ), (**c**) curcumin treated (■) SiHa and in the (**d**) untreated (●), (**e**) menadione treated (Δ), (**f**) curcumin treated (■) HCT116 cells. Values are expressed as mean ± SD, n = 3.

Curcumin did not significantly affect both the intracellular antioxidant enzymes under consideration, whereas it significantly affected both intracellular superoxide and hydroxyl levels. This leads to the question whether there is any correlation between the temporal variations of various RS and antioxidant concentrations in the cells. Graphs were plotted between the temporal levels of PSS RS and enzyme levels to investigate a possible dynamic correlation between the two. For one of the most direct RS - antioxidant pairs, superoxide-SOD reported^29,30^, it was observed that there is no apparent correlation, as seen from the coefficient of determination (R^2^) for a linear relationship between the pseudo steady-state levels of SOD and superoxide radical levels (Fig. 5) in either the untreated or the drug-treated cells. A similar lack of correlation was observed in other systems such as a microalga, *Chlorella vulgaris*, and a bacterium, *Bacillus subtilis* (Supplementary Figs. 1, 2). This lack of correlation between the dynamic levels of RS and its scavenging enzyme suggests that the individual antioxidant values are not surrogate measures even for ‘their’ RS. Also, as mentioned in the introduction the characteristic time constants of RS reactions and the relevant antioxidant enzyme synthesis through transcription-translation are different by many orders of magnitude. Therefore, the use of antioxidants as markers of cellular stress levels may need a re-consideration. The above results also suggest that the use of antioxidant rhythm in therapy may be inappropriate as manipulation of temporal variations of antioxidants might not be able to induce the desired changes to the RS levels in cells. Instead, if a time-profile of RS in the relevant tissues can be generated, the antioxidant intake can be temporally designed to coincide with high RS levels to ensure effectiveness.

### Entrainment of redox rhythms in menadione and curcumin treated SiHa and HCT116 cells

Analysis of the temporal variations of RS levels in untreated and drug treated cells was done by using Lomb Scargle Periodogram (LSP) method in PAST software, to determine the periodicity of the data (Table 1), as mentioned in Materials and Methods. It was observed that there is an endogenous superoxide rhythm of about 15.4 h and 23 h respectively in the untreated SiHa and HCT116 cells. Interestingly, both menadione and curcumin induced entrainment (reset) of these endogenous rhythms to a shorter period. The curcumin induced reset was more pronounced with changes from 15.4 h to 9.6 h in SiHa and 23 h to 8.2 h in HCT116 as compared to menadione induced reset from 15.4 h to 11 h in SiHa and 23 h to 12 h in HCT116 (Supplementary Fig. 3). Also, this improved reset of rhythms by curcumin occurred at lesser drug concentrations as compared to menadione in both the cell lines.

**Table 1:** Changes in cytotoxicity and RS rhythms^†^ for different time points of drug addition.

Time of drug addition	Parameter	Untreated control		curcumin		menadione	
		SiHa	HCT116	SiHa	HCT116	SiHa	HCT116
0 h drug addition	superoxide rhythm period (h)	15.38	22.59	9.62	8.17	10.99	12.00
	Hydroxyl rhythm period (h)	25.00	20.21	11.11	8.90	21.74	15.36
4 h drug addition	superoxide rhythm period (h)	15.38	22.59	9.17	9.17	9.62	12.82
	Hydroxyl rhythm period (h)	25.00	20.21	8.33	9.60	20.41	20.25
8 h drug addition	superoxide rhythm period (h)	15.38	22.59	12.05	—*	12.35	12.35
	Hydroxyl rhythm period (h)	25.00	20.21	23.81	10.11	25.00	20.25

^†^Data averaged for n = 3. The rhythm periods are given for p < 0.01 by LSP analysis using the software PAST. The power spectra with individual p values are given in the Supplementary Information.

*No significant rhythm was found.

Hydroxyl radical levels in both SiHa (25 h) and HCT116 (20.2 h) showed an endogenous, near circadian rhythm (Table 1). Even though no considerable changes in hydroxyl radical levels were observed when the cells were treated with menadione. Interestingly, menadione induced a slight rhythm reset from 25 h to 21.74 h in SiHa and 20.2 h to 15.4 h in HCT116. The curcumin treated cells showed a larger hydroxyl radical rhythm reset from 25 h to11.1 h in SiHa and 20.2 h to 8.9 h in HCT116 (Supplementary Fig. 4). The higher efficacy of curcumin as compared to menadione combined with the larger reset of both hydroxyl and superoxide rhythms by the drug implies a correlation between the RS rhythm reset and the cytotoxic effects of the drugs.

**Figure 6:**
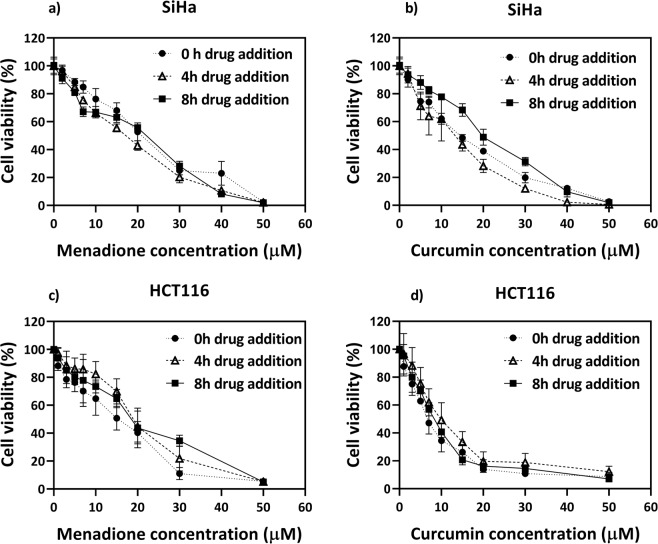
The cytotoxic effects of (**a**) menadione, (**b**) curcumin depend on the time point of drug addition in SiHa and (**c**) menadione, (**d)** curcumin depends on the time point of drug addition in HCT116. The cells were seeded at 1 × 10^4^ cells per well in 96 well plates in serum free DMEM. Medium change to DMEM with 10% serum, 24 h post seeding is considered to be 0 h. The drugs were introduced at 0, 4 or 8 h post medium change. MTT assay was done 48 h post drug treatment. Data represented as mean ± SD, n = 3.

Menadione and curcumin are both known to induce cytotoxicity in cancer cells by RS generation; RS are important regulators of cellular timekeeping mechanisms. Alterations in the RS rhythms could thus cause changes in multiple pathways in the cell and induce cytotoxicity as there exists crosstalk between the cellular redox and cell cycle regulatory systems. To check if indeed, the cells were differentially sensitive to drug addition at different time points, the cytotoxic effect for drug addition at various time points after the medium change was measured. It was observed that in SiHa, at IC_50_ concentrations, an increase in cytotoxicity of 18% for menadione and 27% for curcumin was present, when the drug was added at 4 h, as compared to drug addition at zero time (Fig. 6a,b). The maximum cytotoxicity was observed when drugs were added at 4 h post medium change in SiHa whereas drug addition along with medium change, i.e., at 0 h gave maximum cytotoxicity in HCT116 (Fig. 6c,d). It can be observed that while menadione induces changes in the rhythm and levels of superoxide radicals in both cell lines, curcumin, the more effective of the two drugs, induces an increase in both superoxide and hydroxyl radical levels as well as reset their rhythms to a higher extent. Also, it is interesting that in menadione treated cells, a lesser hydroxyl rhythm reset is observed for certain time points corresponding to higher cytotoxicities, even though no significant changes in hydroxyl radical levels are found, further implying the involvement of rhythm reset in improving cytotoxic efficacy.

The rhythm reset has also been observed to have a direct correlation to the improved cytotoxicity for drug addition at 4 h in SiHa and 0 h in HCT116 as observed by the higher rhythm reset (Supplementary Fig. 5). The superoxide rhythm reset is comparatively lesser for both drugs when added at 8 h (Supplementary Fig. 6). Interestingly, we observe the complete loss of rhythms for both the RS when the drugs are added at 12 h post medium change. This is also observed in the case of hydroxyl radicals in curcumin treated HCT116 cells for drug treatment at 8 h. This could be because of interference from other cellular clock machinery or rhythms and needs to be explored further.

Therefore, the endogenous rhythms of RS seem to affect the drug efficacy and they could be used as a generic target for improved cancer therapy. Since RS are important signaling molecules in the cell interacting with multiple signaling networks, manipulation of RS rhythms for improved drug tolerance and efficacy has potential use in multiple systems. However, further in-depth studies with other drugs and cell lines, as well as *in vivo* studies need to be performed before RS rhythms could be used as a target for cancer chronotherapy.

## Conclusions

Two redox mediated anticancer compounds, menadione and curcumin, were shown to induce the superoxide and hydroxyl radicals differently in SiHa, a human cervical cancer cell line and HCT116, a human colon cancer cell line. Also, they differently entrained (reset) the rhythms of these RS. The rhythm reset was found to correlate to the drug efficacy. The cytotoxic effect of the drug was higher where RS rhythm reset was higher. Further, we observed a difference in the efficacy of the drugs upon drug addition at different points of the endogenous RS rhythm; a 27% higher efficacy in SiHa with curcumin, when added at 4 h compared to the addition at 0 h demonstrates the significance of this finding in a cancer treatment context. In addition, lack of correlations between the dynamic levels of RS and its corresponding antioxidant in the untreated or drug treated cells were observed, which suggests that the use of antioxidant levels as indirect markers of cellular stress levels may need a re-consideration.

## Materials and Methods

### Culture growth

SiHa and HCT116 cells were maintained in complete DMEM (Himedia, India). To the DMEM, 1x of penicillin/streptomycin (Himedia, India) and 10% heat-inactivated fetal bovine serum (FBS; Invitrogen, USA) were added. Cells were cultured in a humidified incubator at 37 °C, 5% CO_2_, and passaged after 80–90% confluence. Before seeding for an experiment, the viable cell concentrations were measured by Trypan blue (Life Technologies, USA) exclusion method in a hemocytometer placed under 10X magnification of an inverted microscope (Eclipse TS100, Nikon, Japan).

### Determination of cytotoxicity of menadione and curcumin

Seeding density of 1 × 10^4^ cells/well were done in a serum-starved condition for synchronization in a 96 well microplate. After 24 hours of seeding, the medium was discarded and 100 µl of fresh DMEM (10% FBS) with different concentrations of menadione or curcumin was added and further incubated for 48 h. MTT (3-(4, 5-dimethylthiazol-2-yl)-2, 5-diphenyl tetrazolium bromide; Himedia, India) at 5 mg/ml in sterile PBS was further diluted in a ratio of 1:10 in a complete medium and incubated for 3 h, after which time the medium was replacd with 100 µl of DMSO to dissolved the formazan crystals. Dual wavelength absorbance measurements at 650 and 570 nm were made using a microplate reader (Model 680, Bio-Rad Laboratories), and the former value was subtracted from the latter to quantitate the cell viability in the wells. To determine the percentage of cell viability at each drug concentration, the percentage ratio of the respective final absorbance values to that of the control (cells treated with 0.1% of DMSO) was calculated.

### Induction of RS by drug treatment

For synchronization, both cell lines were first seeded at 4 × 10^5^ cells/well in 6-well dishes (Nunc) in a serum-starved condition. The medium was replaced with fresh DMEM (10% FBS) after 24 h with predetermined concentration of menadione or curcumin (Sigma Aldrich, USA) for inducing RS inside the cells. The drug addition time was taken as 0 h, and cells were collected by trypsinization every 4 h, until 48 h, one well for each set at each time point. Untreated cells, without drugs, were otherwise maintained at the same culture conditions as of the menadione/curcumin treated cells.

### Intracellular superoxide quantification

Intracellular superoxide concentrations were measured in both cell lines by the fluorescence-based assay using dihydroethidium (DHE; Sigma Aldrich, USA) dye. For specific estimation of hydroxyethidium, the 405/570 nm excitation/emission wavelength pair^31^ was used. To convert the fluorescence unit to the actual concentrations the calibration curve was made as described elsewhere^32^. Catalase (400 U mL^−1^) was also added in the mixture, to remove the hydrogen peroxide generated by the reaction.

For samples collected every 4 h, cells at 2.5 × 10^5^ cells mL^−1^ were suspended in ice-cold PBS containing 20 µM of the cell permeable DHE dye. The cells were incubated at 37 °C for 30 minutes inside humidified incubator, centrifuged at 2,000 rpm and resuspended in 1 ml of PBS. Then further 200 µL of cell suspension per well was aliquoted in the 96 well plate, and fluorescence measurements were taken in a multimode plate reader (Enspire, PerkinElmer, UK). PBS was considered as a blank.

### Intracellular hydroxyl quantification

The measurement of intracellular hydroxyl radical concentrations was quantified by a fluorescent assay using hydroxyphenyl fluorescein (HPF; Life Technologies, USA) dye. To ensure specificity, an excitation/emission wavelength of 480/515 nm was used^33^. For the calibration curve, hydroxyl radicals were generated from hydrogen peroxide using the Fenton reaction. The Fe^2+^ ions required were obtained from a solution of ammonium iron (II)sulfate hexahydrate (Sigma Aldrich, USA) dissolved in 0.01 N HCl.

For sample preparation, cells were suspended in 10 µM of the cell permeable dye HPF, dissolved in ice-cold PBS at 2.5 × 10^5^ cells mL^−1^. Then the cells were incubated for 25 minutes at room temperature in the dark, centrifuged at 2,000 rpm and resuspended in the 1 ml of PBS. Then, 200 µL of cell suspension per well was aliquoted into 96 well plates and fluorescence values were measured in a multimode plate reader (Enspire, PerkinElmer, UK). Blank values for PBS were subtracted from the readings.

### Intracellular SOD quantification

The SOD activity was analysed by using a SOD determination kit (Sigma Aldrich 19160). Control and drug treated cells were collected at different times by trypsin treatment, washed thrice with PBS, and store at −80 °C. Cell lysis was accomplished with a sonicator (Q700, Q-Sonica, USA). After sonication, the cells lysate was centrifuged and then carried forward for assay. The specific (sp) SOD values were determined by normalizing the measured intracellular SOD concentrations with the corresponding cell numbers.

### Intracellular catalase quantification

The cell lysate so obtained for SOD assay described above was assayed for catalase as well by measuring hydrogen peroxide level reduction on treatment with the sample, using the fluorescent dye Amplex Red (Invitrogen, USA). Amplex Red reacts with the unscavenged hydrogen peroxide in the system to form a fluorescent product resorufin, which is specific to an excitation/emission wavelength of 570/585 nm. The sp. catalase values were determined by normalizing the measured intracellular SOD concentrations with the corresponding cell numbers.

### Determination of rhythm parameters

The RS temporal data was processed using the open source software PAST^34^, to check for possible rhythms and to obtain the period of the rhythm, using the spectral analysis tool based on Lomb – Scargle Periodogram (LSP) method. The LSP method uses power spectral analysis to determine the presence of statistically significant rhythms in the given data, assuming a sinusoidal rhythm^35^.

### Statistical analysis

All data retrieved from the three independent experiments were analyzed in a software named GraphPad Prism 7 (GraphPad, San Diego, CA, USA). The same software was used to compile all the graphs with error bar depicting standard deviation ± mean for 3 independent experiments. Statistical analysis for analyzing the rhythm frequency in the temporal RS data was done in PAST. According to the characteristics of experiment, statistical analysis was done by either Student’s t-test or two-way ANOVA in a Graph Pad PRISM 7, and the statistical significance of the data was considered (α = 0.05 and p < 0.05) in both the softwares.

## Supplementary information

Supplementary Information.

## Data Availability

The datasets generated during and/or analysed during the current study are available from the corresponding author on reasonable request.
