# Supplementary material for: Improved redox anti-cancer treatment efficacy through reactive species rhythm manipulation
Source: Sci Rep. 2020 Jan 31;10:1588. doi: 10.1038/s41598-020-58579-2 (PMC6994657; doi:10.1038/s41598-020-58579-2)
Supplement: Supplementary file 1 — Supplementary Information. [file 41598_2020_58579_MOESM1_ESM.pdf]

Supplementary Information

for

Improved redox anti-cancer treatment efficacy through reactive species rhythm manipulation

Uma Kizhuveetil, Sonal Omer, D. Karunagaran, and G.K. Suraishkumar*

Department of Biotechnology, Bhupat and Jyoti Mehta School of Biosciences building

Indian Institute of Technology Madras, Chennai, India-600036

***Correspondence to:**

G.K. Suraishkumar
Department of Biotechnology, Bhupat and Jyoti Mehta School of Biosciences building
Indian Institute of Technology Madras, Chennai 600036 India
Phone: +91 44 2257 4105
Fax: +91 44 2257 4102
E-mail: gk@iitm.ac.in
ORCID ID: 0000-0002-6521-4494

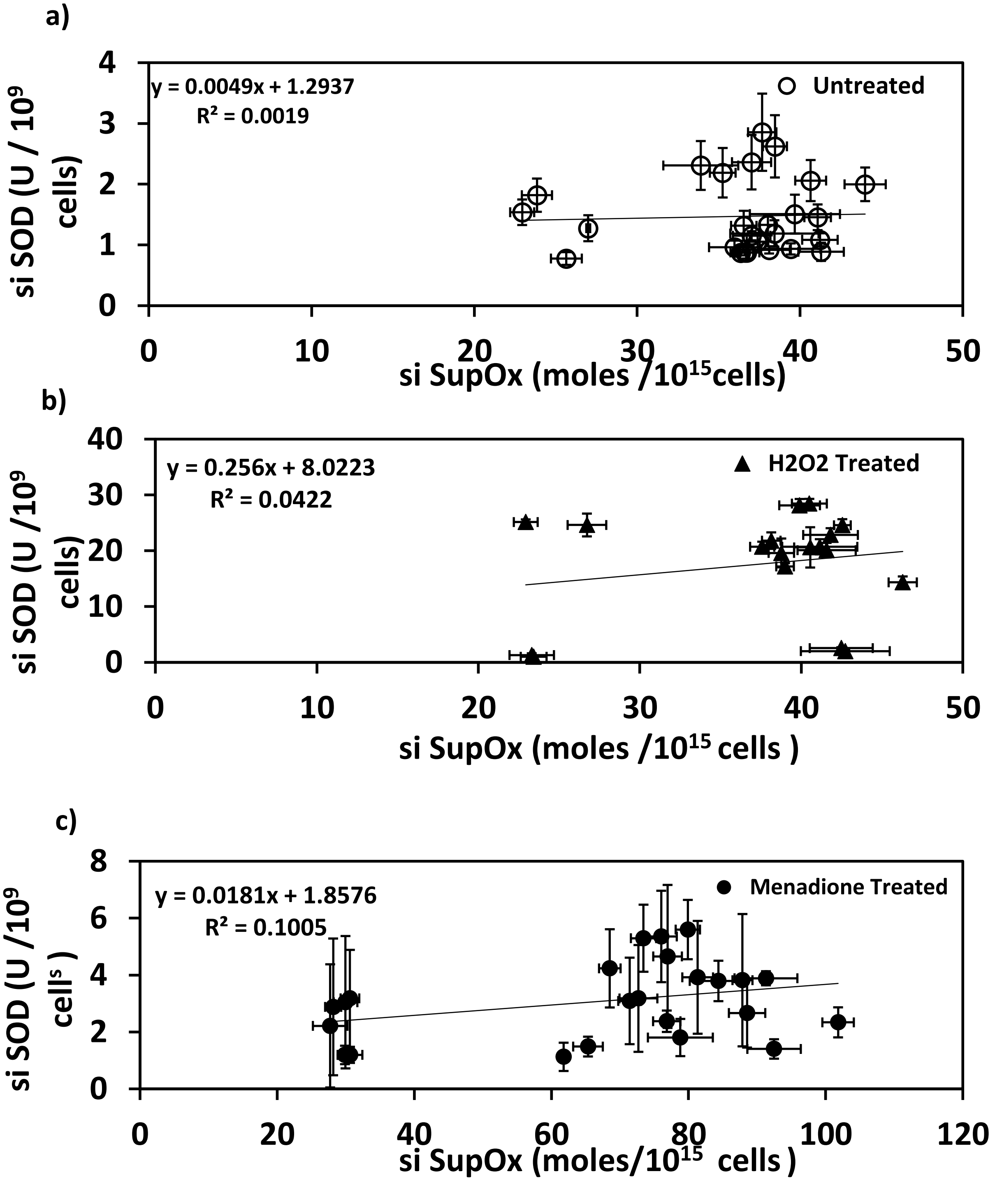

Supplementary Fig. 1: There is no evident correlation between SOD and superoxide levels in the microalga *Chlorella vulgaris* a) untreated control b) H_2O_2 treated cells c) menadione treated cells. Values are expressed as mean ± SD. Data taken from experiments done by Dr. Ranjini Balan and Dr. Steffi Jose.

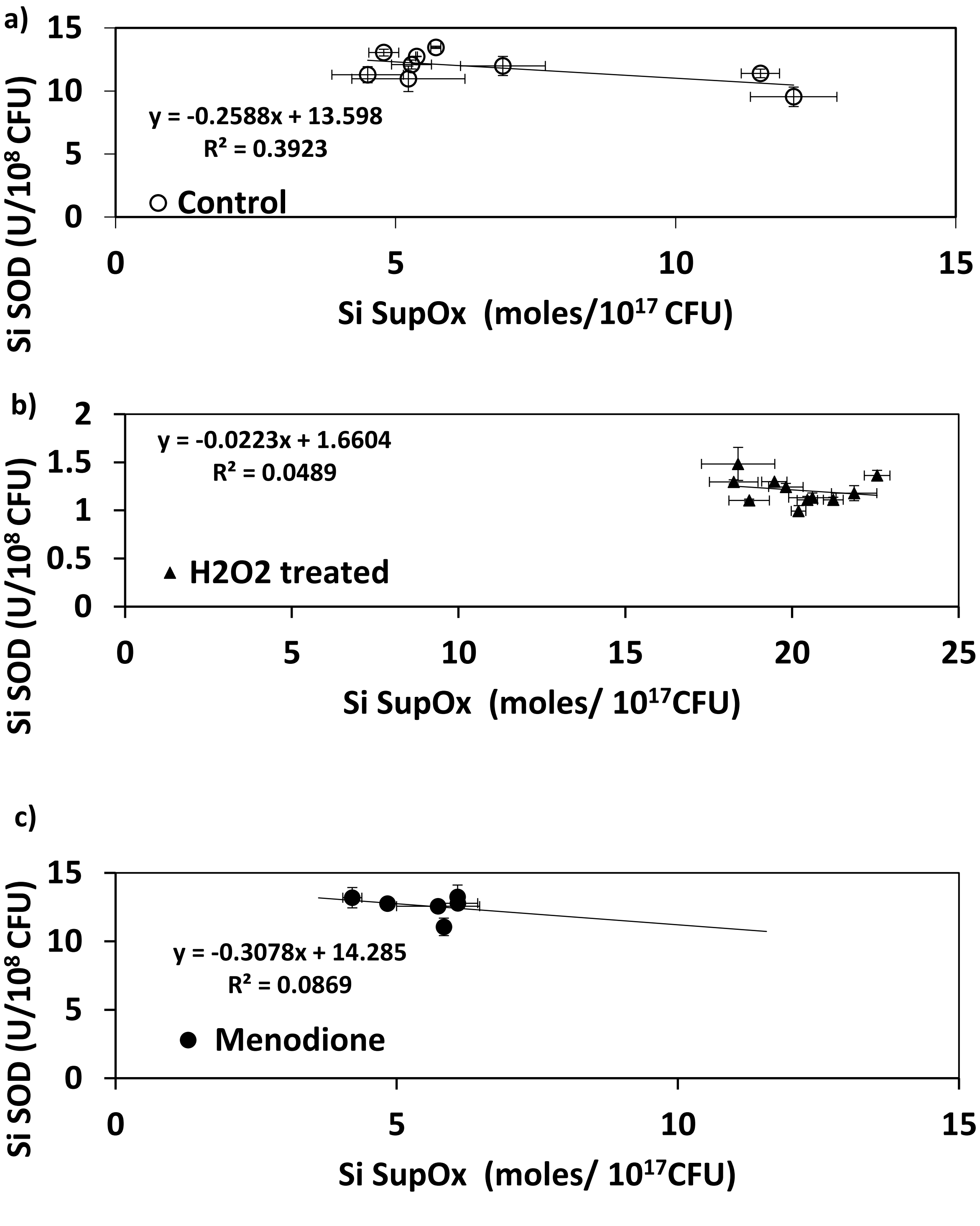

Supplementary Fig. 2: There is no evident correlation between SOD and superoxide levels in the bacterium *Bacillus subtilis* a) untreated control b) H_2O_2 treated cells c) menadione treated cells. Data taken from experiments done by Ms. Archanaa Sundararaghavan and Dr. Steffi Jose.

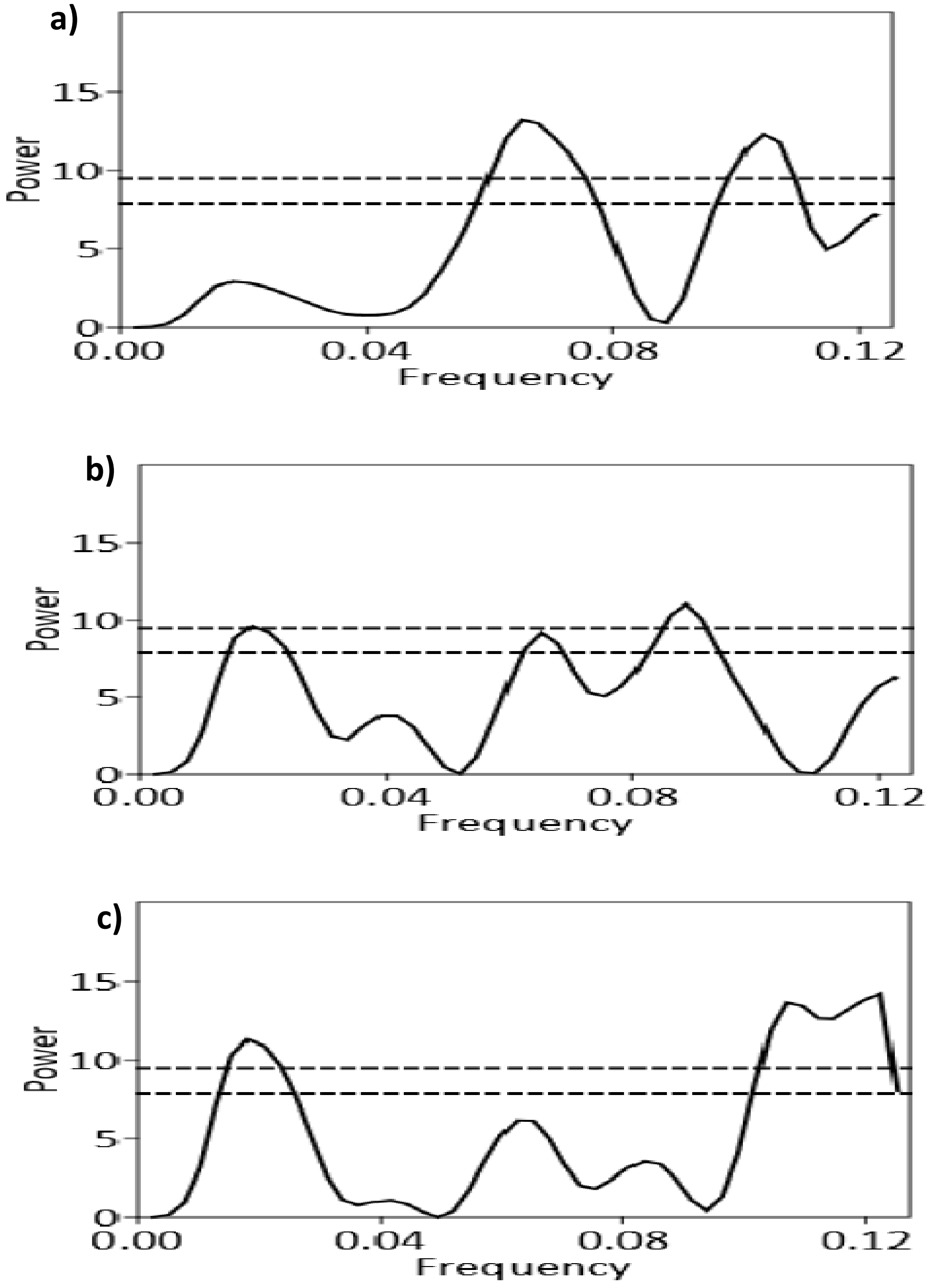

Supplementary Fig. 3: Time series power spectral graphs of frequencies generated in the Lomb–Scargle Periodogram for superoxide in SiHa cells. a) Untreated control, b) menadione treated and c) curcumin treated. The top and bottom dotted lines represent the $p < 0.01$ and $p < 0.05$ levels respectively. The single dotted line represents $p < 0.05$. The frequency with highest power, i.e., the most probable rhythm for the data is considered. The period is calculated as the inverse of the peak frequency.

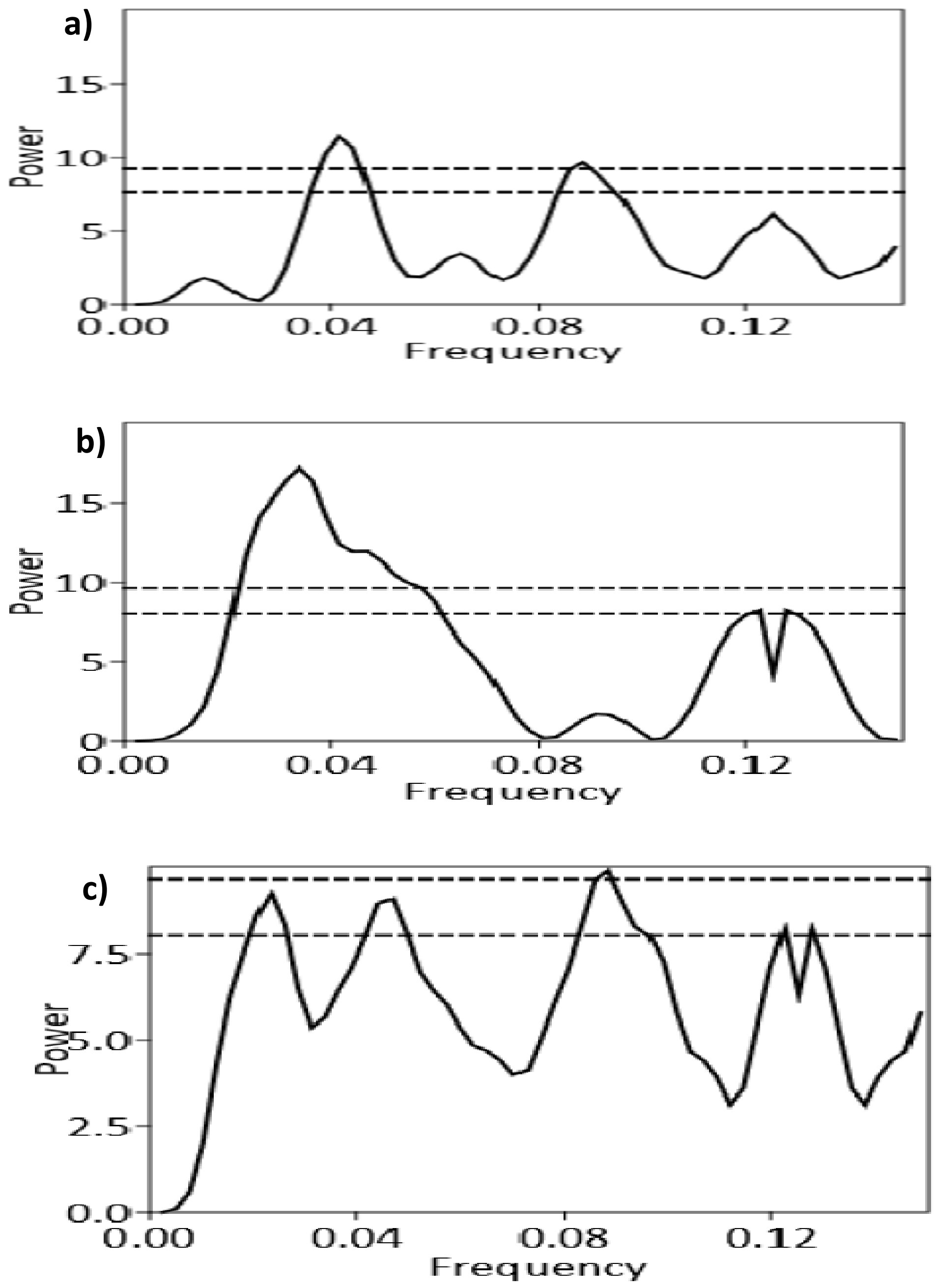

Supplementary Fig. 4: Time series power spectral graphs of frequencies generated in the Lomb–Scargle Periodogram for hydroxyl radicals in SiHa cells. a) Untreated control, b) menadione treated and c) curcumin treated. The top and bottom dotted lines represent the $p < 0.01$ and $p < 0.05$ levels respectively. The frequency with highest power, i.e., the most probable rhythm for the data is considered. The period is calculated as the inverse of the peak frequency.

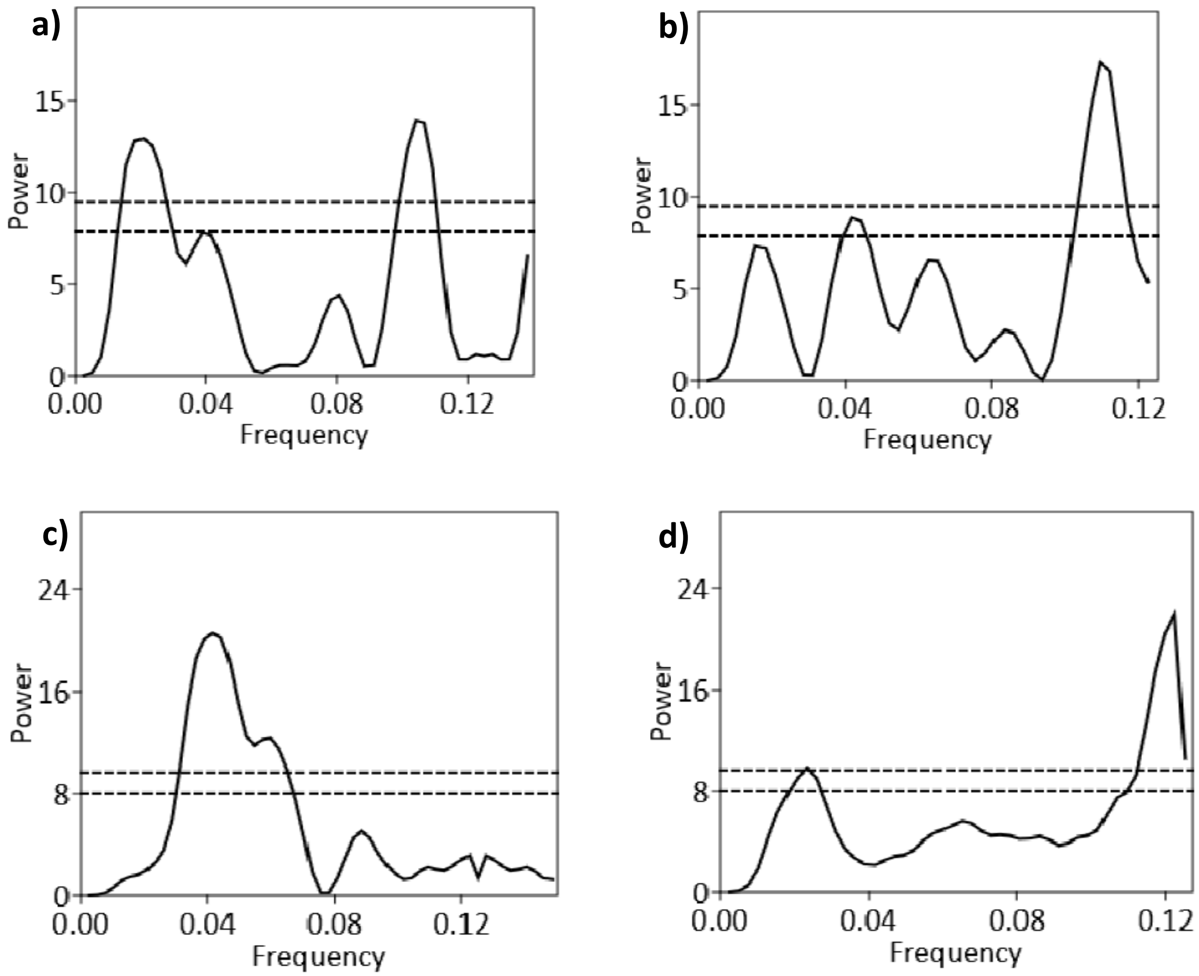

Supplementary Fig. 5: Time series power spectral graphs of frequencies generated in the Lomb–Scargle Periodogram for drug addition at 4h in SiHa cells. a) Superoxide in menadione treated cells, b) superoxide in curcumin treated cells, c) hydroxyl in menadione treated cells, d) hydroxyl in curcumin treated cells. The top and bottom dotted lines represent the $p < 0.01$ and $p < 0.05$ levels respectively. The frequency with highest power, i.e., the most probable rhythm for the data is considered. The period is calculated as the inverse of the peak frequency.

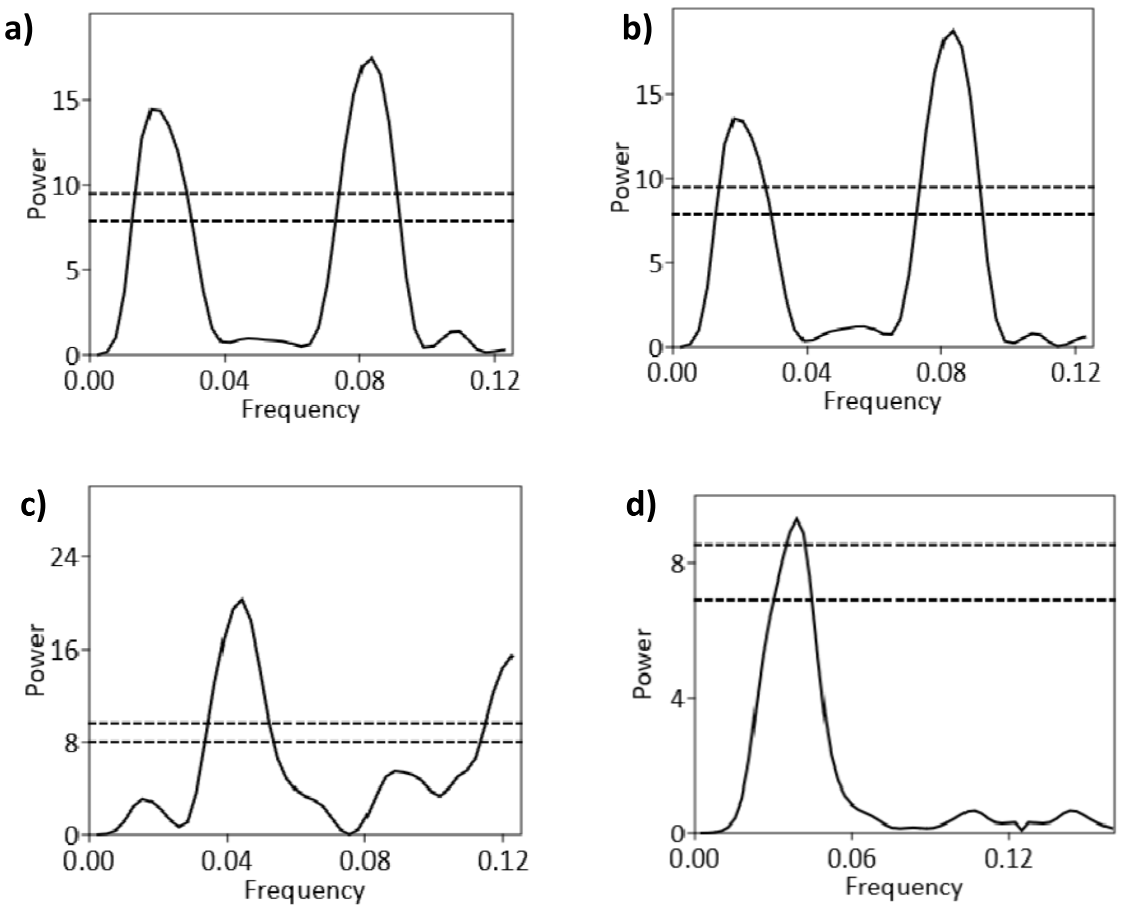

Supplementary Fig. 6: Time series power spectral graphs of frequencies generated in the Lomb–Scargle Periodogram for drug addition at 8h in SiHa cells. a) Superoxide in menadione treated cells, b) superoxide in curcumin treated cells, c) hydroxyl in menadione treated cells, d) hydroxyl in curcumin treated cells. The top and bottom dotted lines represent the $p < 0.01$ and $p < 0.05$ levels respectively. The frequency with highest power, i.e., the most probable rhythm for the data is considered. The period is calculated as the inverse of the peak frequency.

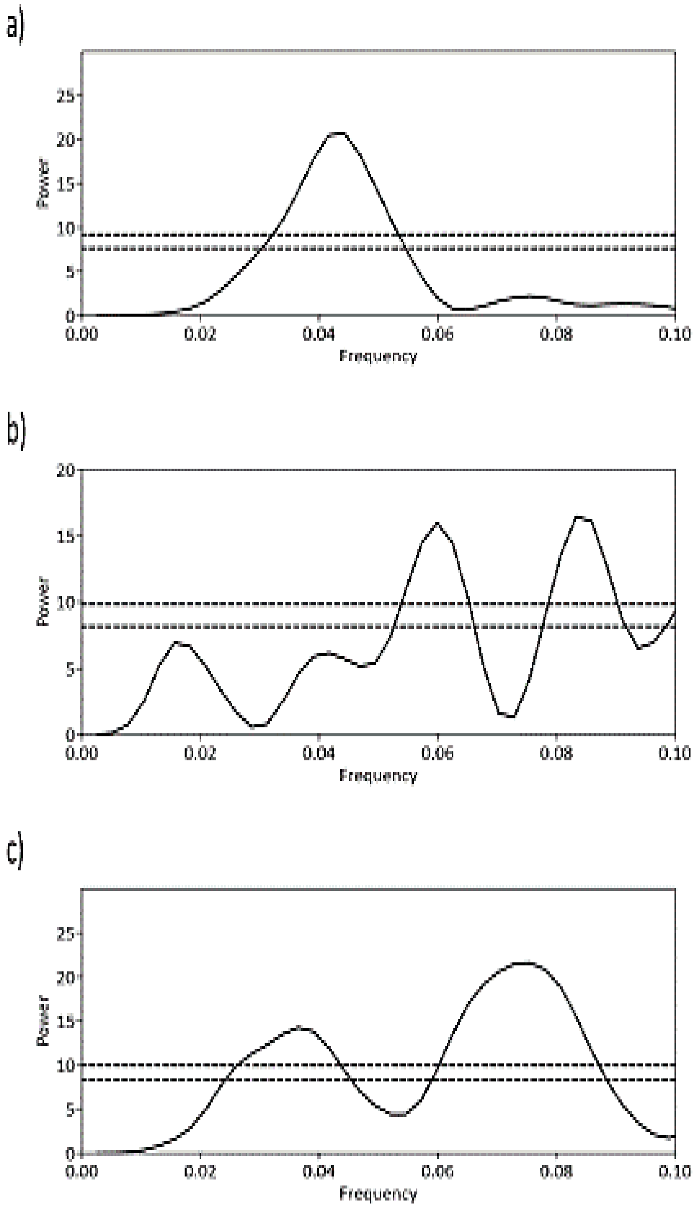

Supplementary Fig. 7: Time series power spectral graphs of frequencies generated in the Lomb–Scargle Periodogram for superoxide in HCT116 cells. a) Untreated control, b) menadione treated and c) curcumin treated. The top and bottom dotted lines represent the $p < 0.01$ and $p < 0.05$ levels respectively. The single dotted line represents $p < 0.05$. The frequency with highest power, i.e., the most probable rhythm for the data is considered. The period is calculated as the inverse of the peak frequency.

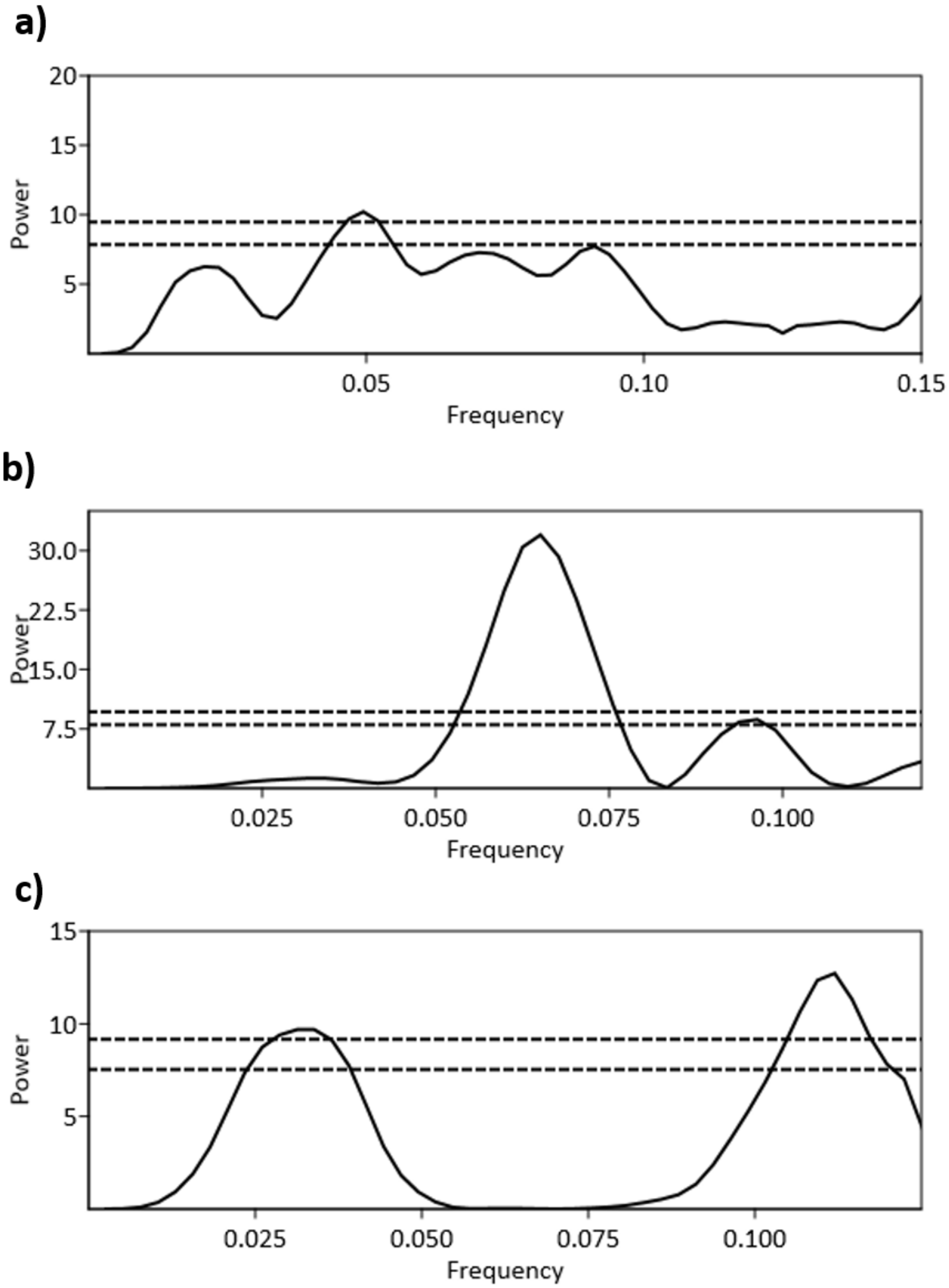

Supplementary Fig. 8: Time series power spectral graphs of frequencies generated in the Lomb–Scargle Periodogram for hydroxyl radicals in HCT116 cells. a) Untreated control, b) menadione treated and c) curcumin treated. The top and bottom dotted lines represent the $p < 0.01$ and $p < 0.05$ levels respectively. The frequency with highest power, i.e., the most probable rhythm for the data is considered. The period is calculated as the inverse of the peak frequency.

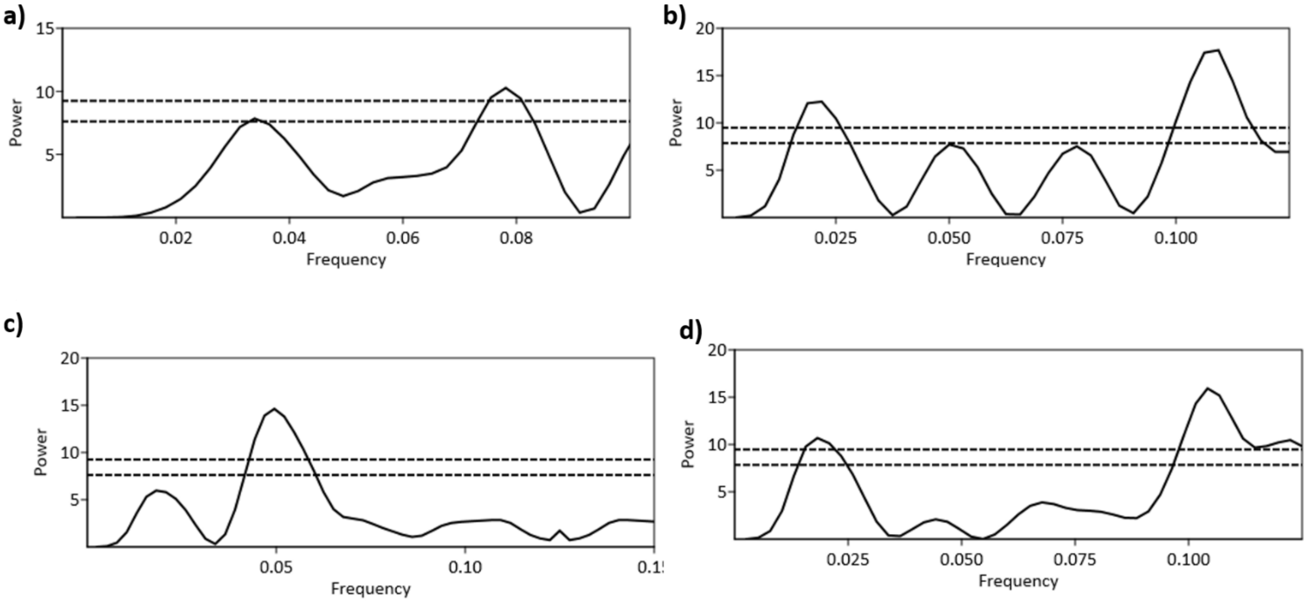

Supplementary Fig. 9: Time series power spectral graphs of frequencies generated in the Lomb–Scargle Periodogram for drug addition at 4h in HCT 116 cells. a) Superoxide in menadione treated cells, b) superoxide in curcumin treated cells, c) hydroxyl in menadione treated cells, d) hydroxyl in curcumin treated cells. The top and bottom dotted lines represent the $p < 0.01$ and $p < 0.05$ levels respectively. The frequency with highest power, i.e., the most probable rhythm for the data is considered. The period is calculated as the inverse of the peak frequency.

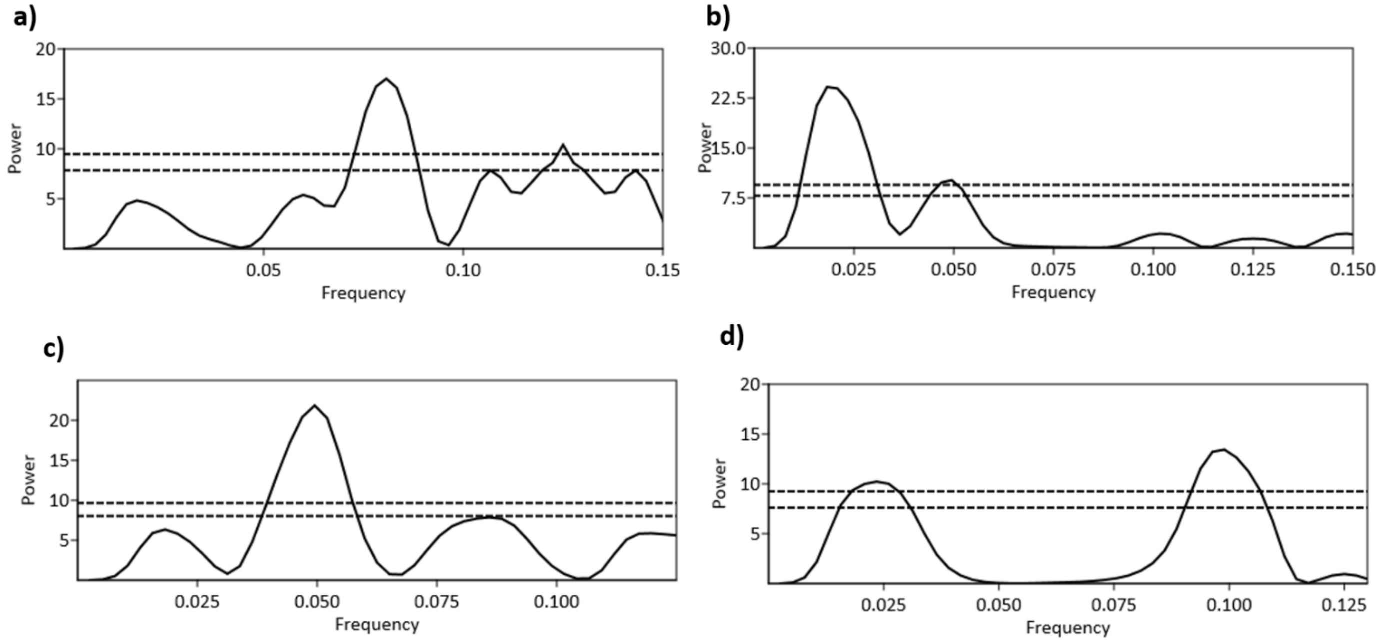

Supplementary Fig. 10: Time series power spectral graphs of frequencies generated in the Lomb–Scargle Periodogram for drug addition at 8h in HCT 116 cells. a) Superoxide in menadione treated cells, b) superoxide in curcumin treated cells, c) hydroxyl in menadione treated cells, d) hydroxyl in curcumin treated cells. The top and bottom dotted lines represent the $p < 0.01$ and $p < 0.05$ levels respectively. The frequency with highest power, i.e., the most probable rhythm for the data is considered. The period is calculated as the inverse of the peak frequency.
